# Semi-rational engineering of cellobiose dehydrogenase for improved hydrogen peroxide production

**DOI:** 10.1186/1475-2859-12-38

**Published:** 2013-04-23

**Authors:** Christoph Sygmund, Paul Santner, Iris Krondorfer, Clemens K Peterbauer, Miguel Alcalde, Gibson S Nyanhongo, Georg M Guebitz, Roland Ludwig

**Affiliations:** 1 Vienna Institute of Biotechnology, Department of Food Sciences and Technology, BOKU-University of Natural Resources and Life Sciences, Vienna, Austria; 2 Department of Biocatalysis, Institute of Catalysis, CSIC, 28049 Madrid, Spain; 3 Institute of Environmental Biotechnology, University of Natural Resources and Life Sciences, Vienna, Austria

## Abstract

**Background:**

The ability of fungal cellobiose dehydrogenase (CDH) to generate H_2_O_2_*in-situ* is highly interesting for biotechnological applications like cotton bleaching, laundry detergents or antimicrobial functionalization of medical devices. CDH’s ability to directly use polysaccharide derived mono- and oligosaccharides as substrates is a considerable advantage compared to other oxidases such as glucose oxidase which are limited to monosaccharides. However CDH’s low activity with oxygen as electron acceptor hampers its industrial use for H_2_O_2_ production. A CDH variant with increased oxygen reactivity is therefore of high importance for biotechnological application. Uniform expression levels and an easy to use screening assay is a necessity to facilitate screening for CDH variants with increased oxygen turnover.

**Results:**

A uniform production and secretion of active *Myriococcum thermophilum* CDH was obtained by using *Saccharomyces cerevisiae* as expression host*.* It was found that the native secretory leader sequence of the *cdh* gene gives a 3 times higher expression than the prepro leader of the yeast α-mating factor. The homogeneity of the expression in 96-well deep-well plates was good (variation coefficient <15%). A high-throughput screening assay was developed to explore saturation mutagenesis libraries of *cdh* for improved H_2_O_2_ production. A 4.5-fold increase for variant N700S over the parent enzyme was found. For production, N700S was expressed in *P. pastoris* and purified to homogeneity. Characterization revealed that not only the k_cat_ for oxygen turnover was increased in N700S (4.5-fold), but also substrate turnover. A 3-fold increase of the k_cat_ for cellobiose with alternative electron acceptors indicates that mutation N700S influences the oxidative- and reductive FAD half-reaction.

**Conclusions:**

Site-directed mutagenesis and directed evolution of CDH is simplified by the use of *S. cerev*isiae instead of the high-yield-host *P. pastoris* due to easier handling and higher transformation efficiencies with autonomous plasmids. Twelve clones which exhibited an increased H_2_O_2_ production in the subsequent screening were all found to carry the same amino acid exchange in the *cdh* gene (N700S). The sensitive location of the five targeted amino acid positions in the active site of CDH explains the high rate of variants with decreased or entirely abolished activity. The discovery of only one beneficial exchange indicates that a dehydrogenase’s oxygen turnover is a complex phenomenon and the increase therefore not an easy target for protein engineering.

## Background

**Figure 1:**
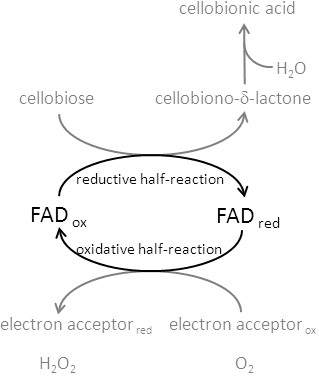
***Half-reactions of the FAD cofactor in the flavodehydrogenase domain of CDH.*** In the reductive half-reaction the oxidized FAD is reduced by e.g. cellobiose, which is converted into the 1,5-lactone before it spontaneously hydrolyzes in the aqueous reaction medium to the aldonic acid. The reduced FAD is regenerated in the oxidative half-reaction by electron acceptors or, very slowly, by oxygen.

The extracellular fungal flavocytochrome cellobiose dehydrogenase (CDH, EC 1.1.99.18) is secreted by wood-degrading, phytopathogenic and saprotrophic fungi [1]. The widespread appearance implies an important function of CDH in the process of wood degradation [2-5]. It is a monomeric protein consisting of two domains [6,7], which oxidizes several carbohydrates at the flavodehydrogenase domain carrying an FAD cofactor. A smaller, heme *b* containing cytochrome domain is connected via a flexible linker. A typical CDH consists of approximately 800 amino acids. In some ascomycetous CDHs, like the one from *Myriococcum thermophilum* (828 aa) used in this study, a family 1 carbohydrate binding module (CBM1) is additionally attached to the C-terminus. The molecular mass ranges from 85 up to 101 kDa depending on the degree of glycosylation, which can account for up to 20% of the molecular mass [8,9]. Basidiomycetous CDHs show a high specificity for cellobiose and cello-oligosaccharides, whereas some ascomycetous CDHs like *M. thermophilum* CDH have a broader substrate specificity and oxidize also other mono-, di- and oligosaccharides, albeit with lower catalytic efficiency [1,5,9,10]. During the reductive half-reaction the FAD cofactor oxidizes suitable carbohydrates at the anomeric C1 atom into intermediary lactones, which hydrolyze spontaneously to the corresponding aldonic acids (Figure 1). Re-oxidation of the FAD can be performed by either two-electron acceptors (quinones, 2,6-dichloroindophenol, phenoxazine- and phenothiazine dyes) or by one-electron acceptors (polysaccharide monooxygenases, cytochrome *c*, ferricyanide and ferrocenium) or very slowly by oxygen [3,4,9].

Due to its versatile properties CDH has been applied in biosensors for the detection of lactose, glucose and catecholamines, in enzymatic biofuel cells as anode catalyst [8,11], for the production of lactobionic acid [12-14], as well as in biodegradation [15] and bioremediation [16]. A more recent proposed application of CDH has been the *in situ* production of H_2_O_2_ for cotton bleaching [10,17,18]. CDH has the potential to replace the currently used mixture of H_2_O_2_ and NaOCl, which causes damage to the cotton fibres, forms toxic by-products and consumes large amounts of energy and water. In the proposed eco-friendly bleaching system, CDH produces the reactive oxygen species. The suitability of CDH for this approach was demonstrated [18]. In contrast to other proposed biocatalysts like choline oxidase [19] or glucose oxidase [20] CDH can produce H_2_O_2_ by oxidation of a wide range of carbohydrates (cellulose and cellodextrins, galactomannans, lactose, maltose or glucose) which occur in the process (e.g. from starch desizing), are added or generated by cellulolytic enzymes [10]. Similarly, the potential of CDH for medical application was recently demonstrated [21,22]. The main drawback of CDH for these applications is its relatively slow H_2_O_2_ production rate compared to oxidases. CDH with increased oxygen reactivity would combine the mentioned advantages with an increased H_2_O_2_ production. Such a CDH would be very attractive for the pulp & paper industry, cotton-bleaching, consumer applications like laundry detergents or antimicrobial functionalization of medical devices, e.g., catheters.

The modulation of the oxygen reactivity in flavoenzymes is currently an active field of research. However, no definite guidelines exist on how to change a dehydrogenase into an oxidase or vice versa [23,24]. It has been shown that the protein matrix surrounding the flavin cofactor (FAD or FMN) has a great effect on the oxygen reactivity [25]. Therefore, semi-rational protein engineering, which targets amino acid residues in the catalytic-site in close vicinity to the FAD by saturation mutagenesis, was the applied strategy to increase the oxygen reactivity of CDH. Several saturation mutagenesis libraries of the *M. thermophilum cdh* gene for five target residues close to the FAD were constructed by the sequence overlap extension (SOE) method and functionally expressed in *S. cerevisiae*. A robust and easy to use high-throughput screening (HTS) assay was established to select CDH variants for improved H_2_O_2_ production. Finally, the mutated *cdh* gene was recombinantly expressed in *P. pastoris* to prepare sufficient amounts of the CDH variant for kinetic characterization and evaluation of the assay.

## Results and discussion

### Expression of *M. thermophilum* CDH in *S. cerevisiae*

CDH is a secretory glycosylated fungal protein, and expression has so far only been successful in eukaryotic expression systems. During the last years, *P. pastoris* was established as the standard expression system for CDH [5,26-28]. Although it is a powerful host for recombinant protein production it is not considered as the preferred host organism for protein engineering by directed evolution. The lack of reliable episomal vectors along with modest transformation efficiencies, preclude in most of the cases the use of this yeast for such approaches. Indeed, no reports of semi-rational engineering or directed evolution of CDHs are published. Even the possibility to express the sole flavodehydrogenase domain of CDH in the prokaryotic expression system *E. coli*[29] has not triggered engineering studies. This can be explained by the essential role of the cytochrome domain for many applications [8,10,15]. So far, heterologous expression of a full length CDH can only be achieved in eukaryotic expression hosts. Therefore, one goal of this study was to establish *S. cerevisiae* as eukaryotic expression system for CDH, which would allow screening for improved variants of full length CDH. *S. cerevisiae* is one of the most successfully used host organisms for directed evolution of eukaryotic proteins due to high transformation efficiencies, easy genetic manipulation and secretion of the target proteins [30,31]. There are several reports in literature where the expression level of the target protein could be increased by the exchange of the native secretory leader sequence with the α-factor prepro leader from *S. cerevisiae*, even subjecting the corresponding fusion gene to several rounds of evolution for improved secretion [32-35].

**Figure 2:**
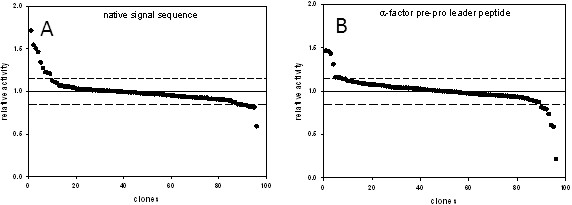
**Landscapes of CDH expression levels.** Dashed lines indicate the variation coefficient. *S. cerevisiae* cells were transformed with plasmid pJRoC30-MtCDH-nat (**A**) and pJROC30-MtCDH-α (**B**). 96 individual colonies of each transformation were cultivated under inducing conditions. Centrifuged supernatants were used for the DCIP-based assay.

Therefore we evaluated in a preliminary experiment the influence of the signal sequence on the expression level of CDH in *S. cerevisiae*. Two nucleotide sequences coding for *M. thermophilum* CDH (*r*CDH) with different signal sequences were cloned into pJRoC30 for expression under the control of the GAL1 promoter. Plasmid pJRoC30-*Mt*CDH-nat encodes the full length CDH including its native secretory leader. In plasmid pJRoC30-*Mt*CDH-α the native signal sequence was replaced by the α-factor prepro leader. The resulting expression vectors were transformed into chemical competent *S. cerevisiae* cells. Ninety-six colonies of each transformation were picked and cultivated in 96-well deep-well plates. After 120 h of induction, supernatants were tested for CDH activity with the (2,6-dichloroindophenol) DCIP assay. The construct with the native secretory leader (0.05 U mL^-1^) showed on average a 3.1 times higher DCIP activity compared to the construct with the α-factor prepro leader (0.016 U mL^-1^). To test the uniformity of the expression levels in the 96-well deep-well plates the coefficient of variation was calculated (Figure 2). Both constructs showed a variation below 15% which is acceptable for screening mutant libraries [36]. However, due to the higher expression levels, the expression cassette employing the native secretory leader was selected for further studies.

### Library construction and high-throughput screening (HTS)

**Figure 3:**
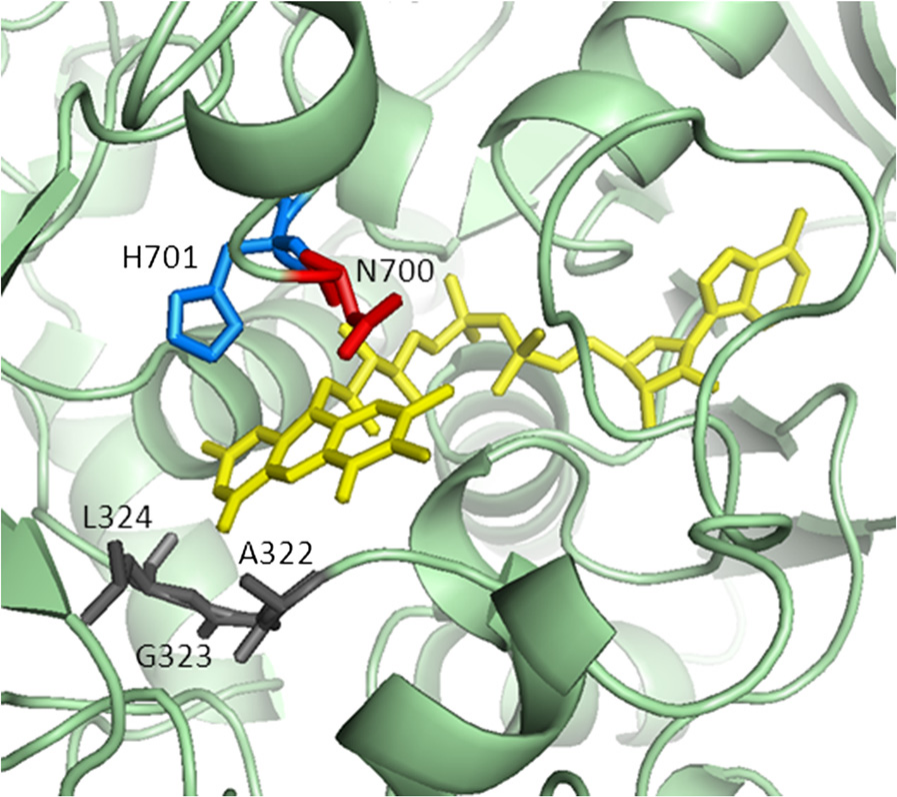
**Model of *M. thermophilum *CDH (template: *P. chrysosporium *CDH structure 1KDG).** The active site with the FAD cofactor (yellow) and five mutated amino acid positions A322, G323, L324 (grey), N700 (red) and H701 (catalytic base, blue) are highlighted.

A comparative (homology) model of CDH from *M. thermophilum* was constructed using the crystal structure of the *Phanerochaete chrysosporium* flavodehydrogenase domain as template (PDB accession code 1KDG, Figure 3). The local alignment of the sequences (see Additional file 1) has a sequence identity of 39% for the modeled flavodehydrogenase sequence (positions 251 – 829), whereas the overall sequence identity is only 35%. The obtained model was carefully tested by local and global model quality check programs. Five amino acids in the catalytic-site close to the FAD were selected for saturation mutagenesis. Three amino acids (G323, A322 and L324) interact with FAD’s isoalloxazine ring system and are in close vicinity of the C4a-N5 locus, which is proposed to be important for the reduction of molecular oxygen in FAD-dependent oxidases. Two amino acids (N700 and H701) are part of the catalytic subsite close to the isoalloxazine ring and fully conserved among CDHs. It was anticipated that mutations of H701, the general base, leads in case of any substitution to inactive variants which can be used to evaluate the mutagenesis method. Degenerated codons of the NNS type were used. The corresponding mutant libraries were double screened for CDH activity and expression with the DCIP-based assay and for H_2_O_2_ production with the ABTS-HRP coupled assay. The ABTS-HRP coupled assay was derived from a published method [18] by switching leucocrystal violet by ABTS and splitting the reaction into a H_2_O_2_ generating part and, after thermal deactivation of the enzyme variants, a colorimetric detection reaction. The DCIP-based assay showed that a large number of mutations at the selected positions result in inactive CDH variants: 55% at G323, 80% at A322, 34% at L324, 29% at N700 and 99% at H701. This demonstrates the importance of the targeted amino acids for enzymatic activity although folding and stability could also be affected. Amino acid H701 corresponds in terms of position to H689 in *P. chrysosporium* CDH which has been proposed to act as catalytic base [7]. Its importance in the catalytic machinery is reflected by the highest number of inactive variants.

During the first screening round twelve variants at position N700 and seven variants at position G232 were selected due to an increased H_2_O_2_ production and subjected to a re-screening. The increased H_2_O_2_ production could be confirmed for all N700 variants in the re-screening while the G232 variants turned out to be false positives. The twelve re-screened variants of position N700 showed an increased DCIP activity (~4 times) as well as an increased H_2_O_2_ production (~5 times) compared to *r*CDH. The sequencing results showed that all of them carried the mutation N700S. The amino acid serine was encoded by any of the 3 possible codons TCT, TCA and AGT, which demonstrates the good performance of the site-saturation mutagenesis method and the reliability of the screening assay.

### Production and purification of *M. thermophilum* CDH variant N700S in *P. pastoris*

**Figure 4:**
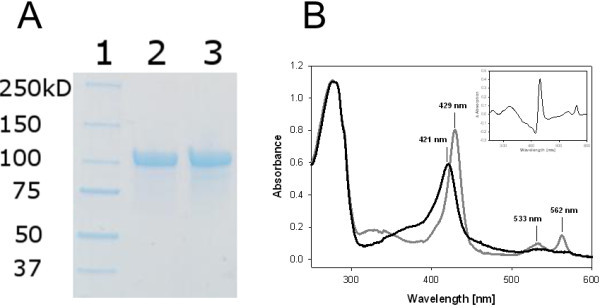
**Verification of protein purity. A**. SDS-PAGE of CDHs expressed in *P. pastoris* after purification. Lane 1, molecular mass marker; lane 2, *r*CDH; lane 3, variant N700S. **B**. Absorption spectra of the CDH variant N700S. The black line shows the oxidized state and the grey line the reduced form. The difference spectrum (red-ox) is shown in the inset.

Although expression levels of CDH in *S. cerevisiae* (0.05 U/mL) were sufficient to perform the screening assay, they were low compared to reported expression levels of CDH using its standard recombinant expression host *P. pastoris*. Therefore we decided to use *P. pastoris* as production host for fermentation to obtain high amounts of CDH for protein purification. The mutation N700S was introduced into the *P. pastoris* expression plasmid pPIC*Mt*CDH by a two-step mutagenesis approach. The resulting plasmid was transformed into electro-competent *P. pastoris* cells. The enzyme was produced with a PCR verified clone in a 7-L stirred and aerated bioreactor. The volumetric CDH activity in the culture supernatant was measured with the DCIP assay and reached a maximum value of 1800 U L^-1^ after 123 h. The recombinant enzyme was purified to apparent homogeneity using a two-step purification protocol (Table 1). Although only the purest fractions were pooled, 75% of the total activity was recovered. The homogeneous preparation consisted of 690 mg CDH (Figure 4A) with a specific DCIP activity of 8 U mg^-1^ and a high absorbance ratio A_420_/A_280_ of 0.54, which was calculated from the UV/Vis absorption spectrum (Figure 4B) and is an indicator for the purity of the CDH sample [1]. The spectrum shows the typical increase of CDH’s *b*-type heme α- and β-band at 562 and 533 nm, respectively, the red-shift from 421 to 429 nm of the heme’s Soret-band and the decrease of the FAD signal in the range of 450 – 500 nm upon reduction of CDH with a carbohydrate substrate.

**Table 1:** Purification scheme of CDH variant N700S

**Purification step**	**Total activity (U)**	**Total protein (mg)**	**Specific activity (U mg**^**-1**^**)**	**Yield (%)**	**Purification (fold)**
Culture supernatant	7380	3360	2.2	100	1
Phenyl-Sepharose	5980	950	6.3	81	2.9
Q-Source	5540	690	8.0	75	3.6

### Oxygen reactivity

**Figure 5:**
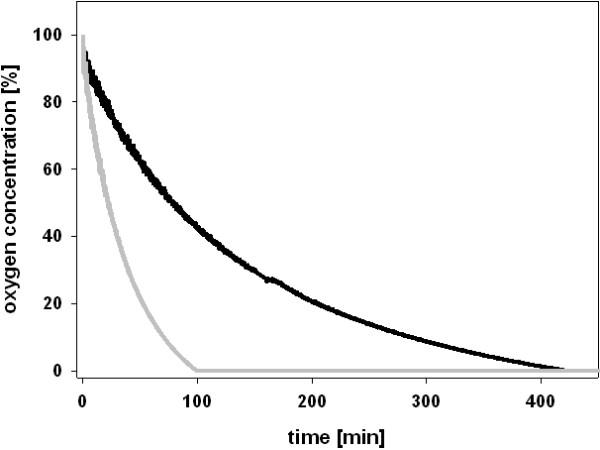
**Measurement of the oxygen consumption rate of *r*CDH (grey line) and N700S (black line).** Reactions were performed in a sealed microreactor containing 30 mM cellobiose in 100 mM McIlvaine buffer, pH 6.0. The decrease of the O_2_ concentration was followed by an inert, luminescent sensor. An excess activity of catalase was added to disproportionate H_2_O_2_.

The O_2_ consumption rates of variant N700S and *r*CDH were determined at 30°C using a fluorescence-based fiber optic sensor. This sensor does not consume O_2_ like a Clark-type electrode, which considerably reduces the oxygen concentration in small reaction volumes and therefore introduces a bias. Both enzymes were applied at the same protein concentration (0.18 mg mL^-1^). Variant N700S consumed all O_2_ present in the 1.9 mL microreactor (2.3 μmol) in 100 min, whereas the unmodified *r*CDH took 420 min (Figure 5). This increased oxygen reactivity was also verified by the stopped ABTS assay in cuvettes. While the fluorescence-based fiber optic sensor measures the decrease in oxygen concentration, the stopped ABTS assay indirectly detects the produced H_2_O_2_. One milligram of *r*CDH produced 0.028 μmol H_2_O_2_ per minute while variant N700S produced 0.127 μmol H_2_O_2_ per minute. Both methods showed that N700S converts O_2_ to H_2_O_2_ about 4.2 to 4.5 times faster than *r*CDH.

### Kinetic characterization of variant N700S

Initial rates of substrate turnover were recorded over a substrate range of 0.003 to 10 mM cellobiose for variant N700S and *r*CDH using DCIP, 1,4-benzoquinone and oxygen as electron acceptor. Kinetic data are summarized in Table 2. The catalytic constant (k_cat_) for cellobiose increased significantly for variant N700S compared to *r*CDH. Without more detailed kinetic measurements it is difficult to discriminate if the mutation increases the rate of the reductive half-reaction (the oxidation of cellobiose and the concomitant reduction of FAD to FADH_2_) or increases the rate of FADH_2_ oxidation by electron acceptors in the oxidative half reaction. The increase of k_cat_ was nearly identical for the two-electron acceptors DCIP (3.0-fold) and 1,4-benzoquinone (3.1-fold) but higher for oxygen (4.5-fold). The K_M_ values for cellobiose increased for N700S when compared to *r*CDH with DCIP (2.9-fold), 1,4-benzoquinone (2.5-fold) and oxygen (2.0-fold). The increased K_M_ is most probably a kinetic effect and not due to changed affinities for the electron acceptors, which have no known binding site. The catalytic efficiencies (k_cat_/K_M_) for variant N700S and *r*CDH are similar for DCIP and 1,4-benzoquinone, but for oxygen the catalytic efficiency of N700S is 2.4-fold higher than for *r*CDH. These data indicate that part of the increased substrate turnover comes from a faster reductive half-reaction, but in the case of oxygen also a faster oxidative half-raction is influencing the overall velocity. In conclusion, position N700 seems to influence both: substrate and oxygen turnover. In the homology model of *M. thermophilum* CDH (Figure 2) the amino acid N700 corresponds to the position of amino acid N688 in *P. chrysosporium*. This asparagine residue is positioned between the catalytic subsite *C* and the binding subsite *B* and with only minor positional changes in the cellobiose molecule it may interact with either of the subsites, thereby influencing substrate binding or catalysis. Due to the close vicinity to the catalytic base H689 it might also influence its proton abstracting properties. The comparative model of variant N700S shows that the serine side chain can easily flip its orientation between both subsites. To fully elucidate the influence of the mutation on the reaction mechanism more elaborate steady-state and presteady-state experiments are needed. The high substrate concentrations used in technological processes, which are much higher than the K_M_ value of the CDH variant for cellobiose, ensures a 4.5-fold increased turnover of the co-substrate O_2_. Under such conditions the obtained variant can be employed in 4–5 times lower amounts than the wild-type CDH to obtain the same amount of H_2_O_2_.

**Table 2:** **Apparent kinetic constants of *r*CDH and variant N700S**

		**Cellobiose**		
**Electron acceptor**	**Enzyme**	**K**_**m **_**(μM)**	**k**_**cat **_**(s**^**-1**^**)**	**k**_**cat**_**/K**_**m **_**(M**^**-1**^ **s**^**-1**^**)**
DCIP	*r*CDH	11.4 ± 0.7	4.1 ± 0.1	360000
	N700S	33 ± 1	12.4 ± 0.6	375000
1,4-benzoquinone	*r*CDH	22 ± 1	4.3 ± 0.1	185000
	N700S	56 ± 2	13.4 ± 0.5	240000
oxygen	*r*CDH	37 ± 3	0.042 ± 0.003	1100
	N700S	73 ± 6	0.19 ± 0.01	2600

Kinetic constants for cellobiose were determined with DCIP, 1,4-benzoquinone and oxygen as electron acceptors at pH 6.0 and 30°C.

## Conclusions

*S. cerevisiae* along with *E. coli* are the most successfully used host organisms for laboratory evolution and semi-rational protein engineering. The ease of genetic manipulation and its high transformation efficiencies due to the ability to maintain autonomously replicating plasmids facilitate the construction of mutant libraries. The successful expression of CDH under the control of the GAL1 promoter in *S. cerevisiae* offers a possibility to easily produce and screen for genetically engineered CDH variants and maybe also other mutated fungal oxidoreductases, which are needed for various biotechnological applications. The developed HTS assay can be easily adapted for other oxidase (H_2_O_2_ forming) activities. For production of selected variants *P. pastoris* is, however, the more efficient expression host.

The fact that most mutations at the selected positions resulted in inactive or less active CDH variants demonstrates that changes in the vicinity of the flavin have a tremendous impact on enzymatic activity. Out of the five selected amino acids only one mutation resulted in an increased H_2_O_2_ production. Further research towards an higher oxygen reactivity of CDH is definitely required for even more efficient cotton bleaching, laundry detergents or antimicrobial functionalization of medical devices.

## Methods

### Chemicals and vectors

Chemicals were purchased from Sigma, Fluka, Roth or VWR and were of the highest purity available. Primers (the nucleotide sequences are shown in Table 3) were obtained from VBC Biotech (Vienna, Austria). Restriction enzymes and T4-ligase were purchased from Fermentas, Phusion polymerase from New England Biolabs and the yeast expression vector pPICZαA from Invitrogen. The uracil independent and ampicillin resistance shuttle vector pJRoC30 was obtained from the Department of Biocatalysis (CSIC, Spain). Recombinant CDH (*r*CDH) was produced as published [10].

**Table 3:** Nucleotide sequences of primers where N is A/T/G/C and S is C/G

**Primer name**	**Sequence (from 5′ to 3′)**
5MT-*Bam*HI	TATGGATCCATGAGAACTTCTTCTAGACTTATCG
5MT-*Xho*HI	TATCTCGAGCAGAATAACGTTCCAAACACC
3MT-*Xho*HI	TATCTCGAGTTACAAACATTGAGAGTACC
5MT-G323X	AGTCAATGCTNNSCTTTGGTTCAAGCC
3MT-G323X	TTGAACCAAAGSNNAGCATTGACTGCG
5MT-A322X	TACCGCAGTCAATNNSGGTCTTTGGTTCAAGC
3MT-A322X	AACCAAAGACCAGCSNNGACTGCGGTACCTCC
5MT-L324X	AGTCAATGCTGGTNNSTGGTTCAAGCCATATTCTTTGG
3MT-L324X	ACCAGCATTGACTGCGGTACCSNNACCAGCAT
5MT-N700X	TCCTTCTAACAGACGTTCTNNSCACTGGATGGGTACTAAC
3MT-N700X	CCAGTGSNNAGAACGTCTGTTAG
5MT-H701X	AGACGTTCTAACNNSTGGATGGGTAC
3MT-H701X	GTACCCATCCASNNGTTAGAACGTCT
5MT-N700S	CAGACGTTCTTCTCACTGGATGGGTAC
3MT-N700S	AGAACGTCTGTTAGAAGGAGAC

### Strains and media

The protease deficient *S. cerevisiae* strain BJ5465 was from LGC Promochem (Barcelona, Spain). SC drop-out plates (synthetic complete) contained 6.7 g L^-1^ yeast nitrogen base (YNB) without amino acids, 1.92 g L^-1^ yeast synthetic drop-out medium supplement without uracil, 2% (w/v) glucose, 25 mg L^-1^ chloramphenicol and 20 g L^-1^ agar. For the preparation of liquid minimal medium the agar was omitted and glucose was replaced by raffinose. The SG/R-CAA expression medium [37] contained 5 g L^-1^ casein hydrolysate, 9.67 g L^-1^ NaH_2_PO_4_, 6.77 g L^-1^ Na_2_HPO_4_, 2% (w/v) raffinose, 2% (w/v) galactose, 0.5% (w/v) glucose and 3.35% YNB. *P. pastoris* X-33 is a component of the EasySelect *Pichia* Expression Kit from Invitrogen. *P. pastoris* transformants were grown on YPD plates (10 g L^-1^ yeast extract, 20 g L^-1^ peptone, 10 g L^-1^ glucose and 100 mg L^-1^ zeocin) and the Basal Salts Medium (Invitrogen) was used for fermentation. The chemically competent *E. coli* strain NEB 5-alpha was purchased from New England Biolabs and used for maintenance and propagation of plasmids. *E. coli* cells were cultivated in Low Salt LB-medium (10 g L^-1^ peptone from casein, 5 g L^-1^ yeast extract, 5 g L^-1^ NaCl and 25 mg L^-1^ zeocin).

### CDH expression in *S. cerevisiae*

The published plasmid pMt1 [10] was used as template for the amplification of *M. thermophilum* CDH cDNA with two different forward primers (5MT-*Bam*HI and 5MT-*Xho*Ifw) and the reverse primer 3MT-*Xho*I. The resulting nucleotide sequences encoded CDH with and without its native secretory leader sequence. The PCR amplicons were digested with the respective restriction enzymes and ligated into the equally treated shuttle vector pJRoC30 under the control of the GAL1 promotor. The resulting plasmid pJRoC30-*Mt*CDH-nat encoded for the native secretory leader whereas in plasmid pJROC30-*Mt*CDH-α it was replaced by the α-factor prepro leader peptide of *S. cerevisiae*. Both plasmids were transformed into competent *S. cerevisiae* cells using the yeast transformation kit (Sigma). Transformed cells were plated on SC drop-out plates and incubated for 4 days at 30°C. From each transformation 96 colonies were picked and cultured in a 96-well deep-well plate (Ritter) containing 100 μL of minimal media per well. These master plates were sealed with Breathe -Easy film (Diversified Biotech) to prevent evaporation and incubated in a shaking incubator (480 rpm) at 25°C and a relative humidity of 80%. After 48 h, 500 μL of expression medium SG/R-CAA were added to each well and the plates were incubated for additional 120 h. The cultivation was stopped by centrifugation for 5 min at 3000 × g. From each well 50 μL of clear culture supernatant were transferred from the master plate to 96-well plate assays using a pipetting robot (Janus, Perkin Elmer). The volumetric activity was measured with the DCIP-based assay.

### Preparation of libraries and HT-screening

A comparative (homology) model of *M. thermophilum* CDH based on the template of *P. chrysosporium* CDH (1KDG, [7]) was used to select positions for mutagenesis. The model was calculated by the Swiss-Model protein structure homology modeling server [38] accessible via the ExPASy web server and checked by using the ANOLEA mean force potential [39], the GROMOS empirical force field energy [40], the composite scoring function QMEAN [41] and a stereochemistry check [42]. Five amino acids located in close vicinity of the FAD cofactor (A322, G323, L324, N700 and H701) were selected for site-saturation mutagenesis. The plasmid pJRoC30-*Mt*CDH-nat was used as template for the site-saturation PCRs, which allowed the construction of *cdh* libraries containing all possible codons at the targeted position. Randomized NNS codons were used to reduce the bias of the genetic code. Mutants were prepared by the sequence overlap extension method [43]. Two complementary mutagenic oligonucleotide primers were designed for each of the 5 target positions (Table 3). The primers were used together with the flanking primers RMLC-sense and RMLN-antisense [44] to amplify two DNA fragments with overlapping ends. In a subsequent fusion PCR these fragments were assembled. PCR products of the mutated *cdh* gene and flanking regions homologous to the vector were purified by electrophoresis, mixed with the *Xho*I and *Bam*HI linearized vector pJRoC30 (ratio PCR product:vector = 4:1) and transformed into competent cells using the yeast transformation kit. For each of the five target positions a library of 352 clones was screened. Individual clones were picked and cultured under the above-mentioned conditions. Four wells per plate were inoculated with *S. cerevisiae* transformed with pJRoC30-*Mt*CDH-nat as a positive control, 2 wells were inoculated with *S. cerevisiae* transformed with empty pJRoC30 as a negative control and 2 wells were not inoculated at all. After 168 h of incubation the culture supernatants were subjected to the DCIP-based screening assay. Therefore, 50 μL of each well were transferred from the master plate to two replica plates. 150 μL of the respective assay mixture (DCIP-based assay and ABTS-based assay) were added by the liquid-handling-robot. Variants with increased H_2_O_2_ production were selected for rescreening. Each of the selected variants was used to inoculate four wells of a new cultivation plate, which was incubated and screened as described above. Exchanges in the nucleotide sequence of approved hits were checked by sequencing. Therefore, colony PCRs were performed using the primers RMLC-sense and RMLN-antisense and the amplified fragments were sent for sequencing.

### HTS assays for enzymatic activity and H_2_O_2_ production

CDH activity was measured by following the time-dependent reduction of 300 μM 2,6-dichloroindophenol (DCIP) at a wavelength of 520 nm (ϵ_520_ = 6.8 mM^-1^ cm^-1^) in 100 mM McIlvaine buffer, pH 5.5, containing 30 mM cellobiose. The reaction was started by adding 150 μL of the DCIP-based assay solution to 50 μL sample in the well and followed in a temperature controlled plate reader at 30°C for 5 min.

H_2_O_2_ production was measured by a modified 2,2′-azino-bis(3ethylbenzthiazoline-6-sulfonate) (ABTS)-based assay. Originally, this assay quantifies the production of H_2_O_2_ by oxidases through the oxidation of ABTS in the presence of horseradish peroxidase. The formation of the green ABTS cation radical is followed spectrophotometrically at 420 nm (ϵ_420_ = 36 mM^-1^ cm^-1^). However, because CDH can reduce the oxidized ABTS cation radical (like other electron acceptors), which would interfere with the assay, a modification was applied. First, 50 μL of a reaction mixture containing 60 mM cellobiose in 100 mM McIlvaine (citrate-phosphate) buffer, pH 5.5, was added to 50 μL of the sample for the production of H_2_O_2_. The reaction mixture was incubated at 30°C for 4 h before CDH was inactivated at 90°C for 10 min. This procedure does not influence the H_2_O_2_ concentration. The colorimetric reaction was started by the addition of 100 μL of ABTS reagent containing 2 mM ABTS and 5.7 U mL^-1^ peroxidase in 100 mM McIlvaine buffer, pH 5.5. The increase in absorbance was followed by a temperature controlled plate reader at 30°C for 5 min. The stoichiometry for this reaction is two since for one mol of H_2_O_2_ two mol of the green ABTS cation radical are formed. The enzymatic activity is given in units (U), which corresponds to the production of 1 μmol cellobionic acid or 1 μmol H_2_O_2_ per min.

### Heterologous production of variant N700S in *P. pastoris*

The plasmid pPIC*Mt*CDH was used as template for the generation of mutant N700S by a two-step mutagenesis approach using PCR and *Dpn*I [45]. The sequences of the used primers 5*Mt*-N700S and 3*Mt*-N700S are given in Table 3. The mutation was confirmed by sequencing (LGC Genomics, Berlin, Germany). The *Sac*I linearized expression plasmid was transformed into electrocompetent X-33 cells and transformants were selected on YPD zeocin plates (1 mg L^-1^). The integration of the gene was verified by colony PCR. A positive transformant was selected for production in a 7-L fermenter according to Harreither et al. [27].

### Protein purification

The CDH variant N700S was purified by a hydrophobic interaction chromatography (HIC) and anion exchange chromatography (AIEX) according to a published procedure [10]. The purification was monitored by determination of total protein and activity. The purity of the enzyme preparation was verified by SDS-PAGE. The homogeneous CDH solution was sterile filtered, aliquoted and stored at −80°C for characterization.

### Molecular properties

SDS-PAGE was carried out using Mini-PROTEAN TGX precast gradient gels (4 – 15%) and Bio-Safe Coomassie for staining (Bio-Rad Laboratories). Unstained Precision Plus Protein Standard was used for mass determination. All procedures were done according to the manufacturer’s recommendations (Bio-Rad Laboratories). The spectra of homogeneously purified N700S were recorded at room temperature from 250 to 600 nm in both the oxidized and reduced state using a U-3000 Hitachi spectrometer (Tokyo, Japan). Spectra were recorded before and shortly after the addition of lactose to the cuvette. The oxidized spectrum was used for determining the purity represented by the ratio of A_420_/A_280_.

### Oxygen consumption rates

A luminescence-based fiber optic sensor (PreSens GmbH, Regensburg, Germany) was used to measure O_2_ consumption rates. Oxygen-saturated 100 mM McIlvaine buffer (oxygen concentration ~1200 μM), pH 6.0, containing 30 mM cellobiose was magnetically stirred in a gas-tight, temperature controlled (30°C) glass vial sealed by a septum (total volume 1870 μL). The reaction was started by adding 100 μL of enzyme solution (3.6 mg mL^-1^) through a cannula.

### Kinetic measurements

CDH activity was assayed using 2,6-dichloroindophenol (DCIP, ϵ_520_ = 6.8 mM^-1^ cm^-1^) or 1,4-benzoquinone (ϵ_290_ = 2.224 mM^-1^ cm^-1^) as electron acceptors. The reactions were followed for 180 sec at 30°C in a Lambda 35 UV/Vis spectrophotometer. To assay CDH activity with oxygen as electron acceptor the modified ABTS assay described above was used. The reaction mixture for the production of H_2_O_2_ contained varying cellobiose concentrations (0.003 – 10 mM) dissolved in 100 mM McIlvaine buffer, pH 6.0, and 0.025 mg mL^-1^*r*CDH or 0.01 mg mL^-1^ of N700S. The reaction mixtures were incubated at 30°C and heat inactivated for 5 minutes at 90°C. The color reaction was started by the addition of 100 μL ABTS reagent. Catalytic constants were calculated using nonlinear least-squares regression by fitting the observed data to the Michaelis-Menten equation (Sigma Plot 11, Systat Software, Chicago, IL, USA). The protein concentration in fermentation and electrophoresis samples as well as of purified enzyme preparations was determined by Bradford’s method using bovine serum albumin as standard and a prefabricated assay from Bio-Rad Laboratories (Hercules, CA).

## Competing interests

The authors declare that they have no competing interests.

## Authors’ contributions

CS and RL planned the study and developed the scheme for high-throughput screening and enzyme characterization. MA selected the expression vector, strains and optimized yeast cultivations. PS and IK carried out the construction and screening of the site-saturation libraries, conducted the *P. pastoris* fermentation and purification of N700S. GG and GN measured the oxygen consumption and interpreted the data. CS wrote the first draft of the manuscript. MA and GG revised the manuscript. RL and CP coordinated the study, verified and interpreted results and revised the final manuscript. All authors have read and approved the final manuscript.

## Supplementary Material

Additional file 1 **Local alignment (Clustal X) of *M. thermophilum *and *P. chrysosporium *flavodehydrogenase domains.** Selected positions for mutagenesis are indicated with arrows. Click here for file
