# Supplementary material for: Semi-rational engineering of cellobiose dehydrogenase for improved hydrogen peroxide production
Source: Microb Cell Fact. 2013 Apr 23;12:38. doi: 10.1186/1475-2859-12-38 (PMC3654988; doi:10.1186/1475-2859-12-38)
Supplement: Additional file 1 — Local alignment (Clustal X) of M. thermophilum and P. chrysosporium flavodehydrogenase domains. Selected positions for mutagenesis are indicated with arrows. [file 1475-2859-12-38-S1.pdf]

ADDITIONAL File

```
MtCDH: -FDYIVVGGGAGGIPAADKLSEAGKSVLLIEKGFASTANTGGTLGPEWLEGHDLTRFDVPGLCNQIWVDS : 319
PcCDH: PYDYIIVGAGPGGIIAADRLSEAGKKVLLLERGGPSTKQTGGTYVAPWATSSGLTKFDIPGLFESLFTDS : 302

                                A322 G322 L324
MtCDH: K-GIACEDTDQMAGCVLGGGTAVNAGLWFKPYSLDWDYLFPDGWKYNDVQPAINRALSRIPGTDAPSTDG : 388
PcCDH: NPFWWCKDITVFAGCLVGGGTSVNGALYWYPNDGDFSSSVGWPSSWTNHAPYTSKLSSRLPSTDHPSTDG : 372

MtCDH: KRYYQEGFEVLSKGLAAGGWTSVTANNAPDKKNRTFAHAPFMFAGGERNGPLGTYFQTAKKRNNFDVWLN : 458
PcCDH: QRYLEQSFNVVSQLLKGQGYNQATINDNPNYKDHVFGYSAFDFLNGKRAGPVATYLQTALARPNFTFKTN : 442

MtCDH: TSVKRVIREGGHITGVEVEPFRDGGYEGIVPVTKVTGRVILSAGTFGSAKILLRSGIGPEDQLEVVAASE : 528
PcCDH: VMVSNVVRNGSQILGVQTNDP-TLGPNGFIPVTPK-GRVILSAGAFGTSRILFQSGIGPTDMIQTVQSNP : 510

MtCDH: KDGPTMIGNSSWINLPVGYNLDDHLNTDTVISHPDVVFYD-FYEAWDDPIESDKNSYLESRTGILAQAAP : 597
PcCDH: TAAAALPPQNQWINLPVGMNAQDNPSINLVFTHPSIDAYENWADVWSNPRPADAAQYLANQSGVFAGASP : 580

MtCDH: NIGPMFWEEIVGADGIVRQLQWTAR-----VEGSLGAPNGHTMTMSQYLGRGATSRGRMTITPSLTTIVS : 662
PcCDH: KLN--FWRAYSGSDGFTRYAQGTVRPGAASVNSSLPYNASQIFTITVYLSTGIQSRGRIGIDAALRGTVL : 648

                                                               N700 H701
MtCDH: DVPYLKDPNDKEAVIQGIINLQNALQNVANLTWLFPNSTITPREYVESMVVSPSNRRSNHWMGTNKLGTD : 732
PcCDH: TPPWLVNPVDKTVLLQALHDVVSNIGSIPGLTMITPDVTQTLEEYVD--AYDPATMNSNHWVSSTTIGSS : 716

MtCDH: DGRKGGSAVVDLDTRVYGTDNLFVIDASIFPGVPTTNPTSYIVVAAEHASSRILALPDLEPVPKYGQCGG : 802
PcCDH: P----QSAVVDSNVKVFGTNNLFIVDAGIIPHLPTGNPQGTLMSAAEQAAAKILAL-----------AGG : 771

MtCDH: REWTGSFVCADGSTCEYQNEWYSQCL : 828
PcCDH: P------------------------- : 772
```

Additional file 1: Local alignment (Clustal X) of *M. thermophilum* and *P. chrysosporium* flavodehydrogenase domains. Selected positions for mutagenesis are indicated with arrows.
